# Predictors of adherence and the role of primary non-adherence in antihormonal treatment of breast cancer

**DOI:** 10.1186/s12885-022-10362-4

**Published:** 2022-12-02

**Authors:** Ida Dragvoll, Anna M. Bofin, Håvard Søiland, Gunnar Taraldsen, Monica Jernberg Engstrøm

**Affiliations:** 1 Department of Clinical and Molecular Medicine, Faculty of Medicine and Health Sciences, Norwegian University of Science and Technology (NTNU), Trondheim, Norway; 2 Department of Breast and Endocrine Surgery, St Olav’s Hospital, Trondheim University Hospital, Trondheim, Norway; 3 Department of Research, Stavanger University Hospital, Stavanger, Norway; 4 Department of Clinical Science, University of Bergen, Bergen, Norway; 5 Department of Mathematical Sciences, Faculty of Information Technology and Electrical Engineering, Norwegian University of Science and Technology (NTNU), Trondheim, Norway

**Keywords:** Breast cancer, Antihormonal treatment, Tamoxifen, Aromatase inhibitors, Adherence, Primary non-adherence

## Abstract

**Background:**

Antihormonal treatment for hormone receptor (HR) positive breast cancer has highly beneficial effects on both recurrence rates and survival. We investigate adherence and persistence in this group of patients.

**Methods:**

The study population comprised 1192 patients with HR-positive breast cancer who were prescribed adjuvant antihormonal treatment from 2004 to 2013. Adherence was defined as a medical possession ratio (MPR) of ≥80.

**Results:**

Of the 1192 included patients, 903 (75.8%) were adherent and 289 (24.2%) were non-adherent. Primary non-adherence was seen in 101 (8.5%) patients. The extremes of age (< 40 and ≥ 80 years) were associated with poor adherence. Patients with metastasis to axillary lymph nodes and those who received radiotherapy and/or chemotherapy were more likely to be adherent. Better adherence was also shown for those who switched medication at 2 years after diagnosis. Primary non-adherence seems to be associated with cancers with a good prognosis.

**Conclusion:**

Adherence to antihormonal therapy for breast cancer is suboptimal. Primary non-adherence occurs among patients with a relatively good prognosis. Non-adherent patients tend to terminate their antihormonal therapy in the initial part of the treatment period. Targeted interventions to improve adherence should be focused on the first part of the treatment period.

## Background

Antihormonal treatment is one of the main treatment modalities in the adjuvant treatment of hormone receptor (HR) positive breast cancer. With increasing knowledge, their use has greatly expanded; from initially being used only in the palliative setting [1], tamoxifen is now prescribed for up to 10 years in the curative setting [2]. The efficacy of both tamoxifen [3–5] and aromatase inhibitors (AI’s) [6–8] has been well documented. Despite this, many patients terminate their treatment prematurely or never initiate the treatment at all.

The World Health Organization emphasizes that adherence presumes the active participation and collaboration of the patient in the treatment process [9]. Adherence may be defined as the extent to which the patient takes a medication as prescribed [10]. Primary non-adherence (PNA) is defined as failure of the patient to fill the first and subsequent prescriptions at the pharmacy. Secondary non-adherence (SNA) refers to failure to take the prescribed medication as directed after the first prescription has been collected [11–13]. Persistence describes the time from initiation of therapy to discontinuation or, in more general terms, whether the patient stays on the treatment as prescribed [14, 15].

Awareness of the importance of adherence to medical treatment has increased in recent decades. However, it has been reported that close to 50% of all patients do not adhere to prescribed medical treatment irrespective of diagnosis [16–18]. Poor adherence prevents medications from exerting their full beneficial effect thereby resulting in unnecessary morbidity and mortality [9–11, 14, 18, 19]. In addition to its effect on the individual patient, it also has a vast effect on health economy leading to increased health care expenditures [19–22].

While there is no doubt that antihormonal treatment in breast cancer is highly beneficial, non-adherence continues to be a challenge. Varying rates of non-adherence to antihormonal treatment have been reported, ranging from 10.8% [23] to 55% [24]. Different definitions of adherence and varying methodological approaches to measuring adherence account for this wide range [14, 25, 26]. Poor adherence to antihormonal treatment has been shown to negatively affect recurrence rates and mortality, and also leads to increased medical costs and reduced quality of life [4, 8, 20, 27, 28].

Side effects of antihormonal treatment is one of the strongest predictors of poor adherence to antihormonal treatment [24, 27, 29–31]. Therefore, identifying patients at risk of poor adherence prior to the onset of the treatment provides an opportunity for early intervention. Predictors of adherence including patient characteristics, tumour characteristics, type of antihormonal treatment and other adjuvant treatment modalities, have previously been studied, however, the results are not entirely concordant. Age seems to be an important factor affecting adherence. A number of studies have shown associations between poor adherence and old age [27, 32], younger age [20, 33], and both [29, 34, 35]. In general, adherence seems to improve with increasing severity of disease [27, 36]. While anastrozole has been shown to be associated with better adherence than tamoxifen by some [6], others have failed to demonstrate a similar association [35]. While acknowledging previous research on adherence to antihormonal treatment, further knowledge on how to best identify patients at risk of poor adherence is needed.

The aim of this study was to examine adherence, and specifically PNA, in a series of Norwegian patients with breast cancer. All patients were diagnosed pre-operatively with HR-positive breast cancer and subsequently underwent surgery at St Olav’s Hospital, Trondheim University Hospital, Trondheim, Norway. All patients were prescribed adjuvant tamoxifen or an AI, either as monotherapy or as a switch regimen. Associations with age, tumour characteristics, nodal status, radiation therapy and chemotherapy were studied as putative factors affecting adherence. Non-adherent patients were subclassified into primary non-adherent or secondary non-adherent. Furthermore, persistence was calculated for these patients.

## Materials and methods

### Study population

**Fig. 1:**
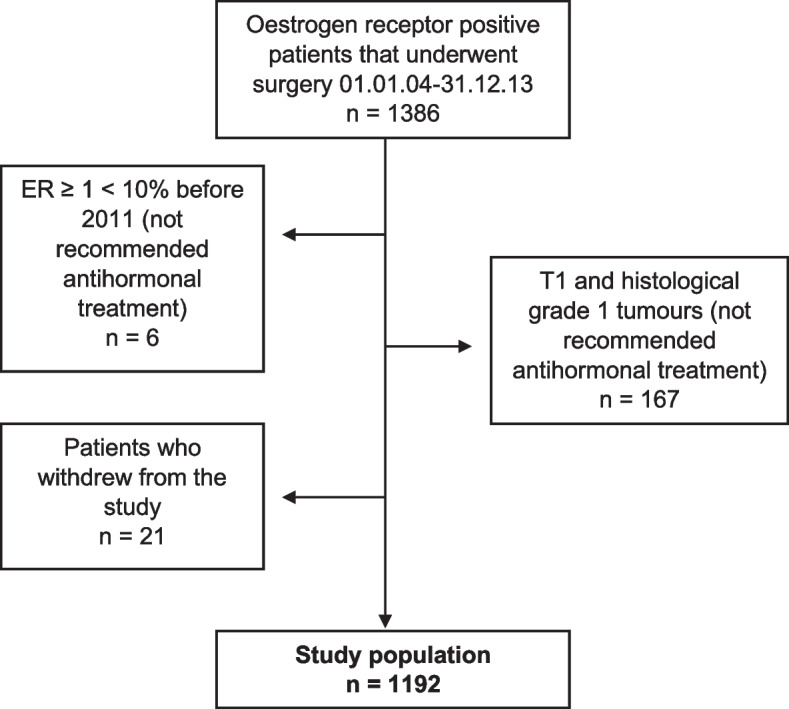
Overview of included and excluded patients

The study population comprised all patients with HR-positive breast cancer who underwent surgery at St Olav’s University Hospital in the period 01.01.2004 to 31.12.2013 and subsequently were prescribed antihormonal treatment as part of their adjuvant therapy. Patients with histological grade 1 tumours less than 20 mm in diameter (T1) were excluded from the material. These patients were not prescribed antihormonal treatment according to national guidelines in the study period. A total of six patients diagnosed before 2011 had tumours showing oestrogen receptor positivity ≥1 < 10% and were therefore excluded from the study as guidelines prior to 2011 stated that oestrogen positivity < 10% did not warrant antihormonal treatment (Fig. 1). All patients in this study were recommended 5 years of antihormonal treatment according to national guidelines operative at the time of diagnosis.

Data regarding tumour characteristics and treatment were retrieved from the hospital medical records. These data were linked to the Norwegian Prescription Database (NorPD) where pharmaceutical records were collected for all patients. After the data were linked, patient identity was replaced by a serial number. The NorPD includes information regarding each dispensed medication, the month and year it was dispensed, the total number of tablets dispensed each time, gender, year of birth, and month and year of death. Data in NorPD covered the time period from 01.01.2004 through 31.12.2019. The long follow-up time ensured at least 5 years of follow-up for all patients. All patients had a one-year follow-up appointment at the surgical out-patient clinic. Thereafter, yearly follow-up by their general practitioner for 10 years. Patients with metastasis at time of primary diagnosis were followed more closely by an oncologist.

### Outcomes

Adherence was determined by the medical possession ratio (MPR). MPR is a recognized and commonly used method to estimate adherence [21, 26]. It is determined by the number of days a medication is at hand within a given time interval [10, 11, 37]. We used the total amount of tablets dispensed as the nominator, this equals the number of days of treatment as the tablets, regardless of type of antihormonal treatment, are taken once daily. The denominator was 5 years for all patients except for those who died during the five-year treatment period. The MPR for patients who died during their five-year course of antihormonal treatment was also included. MPR for these patients were calculated based on the length of time they were alive after commencing the treatment. Patients were considered adherent to the given treatment if MPR reached 80% or more. This cut-off is widely used for differentiating between adherence and non-adherence [37–39]. Adherence was correlated to tumour characteristics (tumour stage, histopathological type and grade, nodal status) and treatment modalities (neoadjuvant treatment, type of antihormonal treatment, radiation therapy, chemotherapy). Persistence was calculated from the date of the first prescription to the date when the supply of the last prescription ended. Pauses in antihormonal treatment of greater than 180 days were regarded as discontinuation of the treatment. Primary non-adherence was defined as failure of the patient to fill the first and subsequent prescriptions of antihormonal treatment.

### Statistical analysis

The differences between subgroups were calculated using logistic regression. Odds ratios (OR) with 95% confidence intervals (95% CI) were calculated. In the univariate analysis we included all clinicopathological data. Predictors of adherence were age, tumour stage, histopathological type, histological grade, axillary lymph node metastasis, neoadjuvant treatment, type of antihormonal treatment, switch of antihormonal treatment at 2 years, radiation therapy and chemotherapy. Furthermore, adherence was predicted as a function of age by including a second-degree polynomial age term in the logistic regression. This term was also used when adjusting for the effect of age. Statistical analyses were performed using SPSS version 28 and Stata version 17.0.

## Results

A total of 1386 patients with HR-positive breast cancer underwent surgery during the study period (Fig. 1). Of these, 22 were diagnosed with breast cancer twice within the study period. In these cases, the first cancer was chosen. Patients with T1, histological grade 1 tumours were excluded (*n* = 167 patients). Six patients diagnosed before 2011 had tumours with oestrogen receptor positivity of ≥1 ≤ 10% and were excluded. Twenty-one patients opted not to participate in the study and were therefore excluded. This leaves a total study population of 1192 patients (1182 females and 10 males).

During the five-year follow-up, a total of 903 patients (75.8%) were adherent (MPR ≥ 80%) to the antihormonal treatment they were prescribed (Table 1). Two hundred and eighty-nine (24.2%) patients were classified as non-adherent. Of these, 101 patients never initiated the treatment and were classified as PNA. A total of 659 (55.3%) had a MPR rate of > 100% indicating that they remained on antihormonal treatment for longer than 5 years.

**Table 1:** Patient characteristics and estimated probability of adherence

Characteristics	Adherent/total	Adherent % (95% CI)	Non-adherent/total	Non-adherent % (95% CI)
	903/1192	75.8 (73.2-78.1)	289/1192	24.2 (21.8-26.8)
Age at diagnosis				
< 40	36/59	61.0 (47.4-73.5)	23/59	39.0 (26.5-52.6)
40-49	164/195	84.1 (78.2-88.9)	31/195	15.9 (11.1-21.8)
50-59	282/350	80.6 (76.0-84.6)	68/350	19.4 (15.4-24.0)
60-69	269/350	76.9 (72.1-81.2)	81/350	23.1 (18.8-27.9)
70-79	104/138	75.4 (67.3-82.3)	34/138	24.6 (17.7-32.7)
≥ 80	48/100	48.0 (37.9-58.2)	52/100	52.0 (42.8-63.1)
Tumour stage				
T1	508/686	74.1 (70.6-77.3)	178/686	25.9 (22.8-29.6)
T2	275/356	77.2 (72.5-81.5)	81/356	22.8 (18.5-27.5)
T3-T4	28/32	87.5 (71.0-96.5)	4/32	12.5 (3.5-29.0)
Unknown^a^	92/118	78.0 (69.4-85.1)	26/118	22.0 (14.9-30.6)
Histopathological type				
Ductal	712/943	75.5 (72.5-78.1)	231/943	24.5 (21.8-27.3)
Lobular	122/153	79.7 (72.5-85.8)	31/153	20.3 (14.2-27.5)
Other	64/89	71.9 (61.4-80.9)	25/89	28.1 (19.1-38.6)
Unknown	5/7	71.4 (29.0-96.3)	2/7	28.6 (3.7-71.0)
Histological grade				
Grade 1	76/104	73.1 (63.5-81.3)	28/104	26.9 (18.7-36.5)
Grade 2	507/686	73.9 (70.4-77.0)	179/686	26.1 (23.0-29.7)
Grade 3	229/285	80.4 (75.3-84.8)	56/285	19.6 (15.2-24.7)
Unknown	91/117	77.8 (69.2-84.9)	26/117	22.2 (15.1-30.8)
Axillary lymph node metastasis				
0	473/665	71.1 (67.4-74.4)	192/665	28.9 (25.6-32.6)
1-3	280/334	83.8 (79.4-87.6)	54/334	16.2 (12.4-20.6)
≥ 4	119/143	83.2 (76.1-88.9)	24/143	16.8 (11.1-23.9)
Unknown	31/50	62.0 (47.2-75.3)	19/50	38.0 (24.7-52.8)
Neoadjuvant treatment				
No	764/1017	75.1 (72.3-77.7)	253/1017	24.9 (22.2-27.7)
Yes	139/175	79.4 (72.7-85.2)	36/175	20.6 (14.8-27.3)
Type of antihormonal treatment				
Tamoxifen only	169/231	73.2 (67.0-78.8)	62/231	26.8 (21.2-33.0)
Tamoxifen ➔ AI	298/343	86.9 (82.8-.90.3)	45/343	13.1 (9.7-17.2)
AI only	284/336	84.5 (80.2-88.2)	52/336	15.5 (11.8-19.8)
AI ➔ Tamoxifen	93/111	83.8 (75.6-90.1)	18/111	16.2 (9.9-24.4)
More than one switch	59/70	84.3 (73.6-91.9)	11/70	15.7 (8.1-26.4)
No antihormonal treatment registered	0/101	0 (0- 3.6)	101/101	100 (96.4-100)
Switch at two years				
No^b^	208/253	82.2 (76.9-86.7)	45/253	17.8 (13.3-23.1)
Yes	245/276	88.8 (84.4-92.2)	31/276	11.2 (7.8-15.5)
Monotherapy	450/562	80.1 (76.5-83.3)	112/562	19.9 (16.7-23.5)
No antihormonal treatment registered	0/101	0 (0- 3.6)	101/101	100 (96.4-100)
Radiation therapy				
No	234/345	67.8 (62.6-72.7)	111/345	32.2 (27.2-37.4)
Yes	669/847	79.0 (76.1-81.7)	178/847	21.0 (18.3-23.9)
Chemotherapy				
No	379/576	65.8 (61.8-69.7)	197/576	34.2 (30.5-38.4)
Yes	524/616	85.1 (82.0-87.8)	92/616	14.9 (12.2-18.0)

^a^108/118 received neoadjuvant therapy. Hormone status was determined on core needle biopsy ^b^Switch at other time point than 2 years (+/− 6 months)

**Fig. 2:**
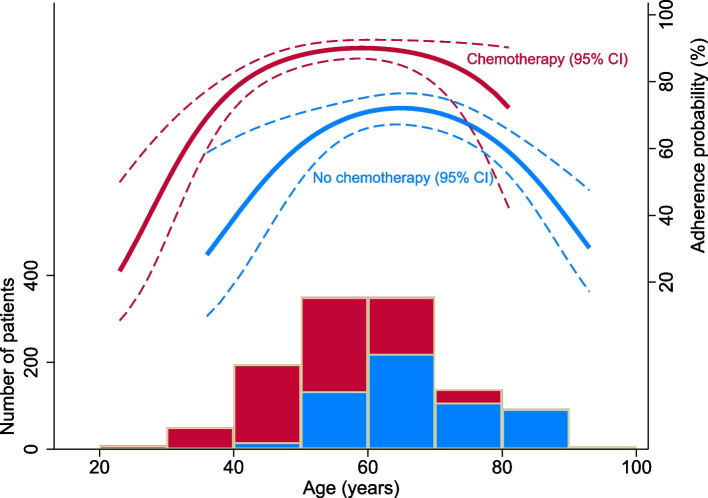
Predicted adherence as a function of age and chemotherapy

The extremes of age (< 40 years and ≥ 80 years) showed significantly lower adherence (OR = 0.4, [95%CI: 0.2-0.7]; *p* = 0.001) and (OR = 0.2, [95%CI: 0.1-0.4]; *p* = < 0.001) respectively compared to patients aged 50-59 years (Table 2). Adherence predicted by a second-degree polynomial age term gives similar results also when grouped by chemotherapy (Fig. 2). Patients with metastasis to axillary lymph nodes as determined by sentinel lymph node biopsy and/or axillary clearance were more likely to be adherent to antihormonal treatment than those without axillary lymph node metastasis, (OR = 2.1, [95%CI: 1.5-3.0]; *p* = < 0.001) for those with 1-3 affected axillary lymph nodes and (OR = 2.0, [95%CI: 1.3-3.2]; *p* = 0.004) for those with ≥4 axillary lymph node metastasis). Similar results were seen when adjusting for age. Patients who switched to an AI (OR = 2.4, [95%CI: 1.6-3.7]; *p* < 0.001) those who received an AI only (OR = 2.0, [95%CI: 1.3-3.0]; *p* = 0.001), and the patients who started their treatment with an AI (OR = 1.9, [95%CI: 1.1-3.4]; *p* = 0.031) were all more likely to be adherent than those who received tamoxifen monotherapy. Those who switched type of antihormonal treatment (tamoxifen to AI or AI to tamoxifen) after 2 years were more likely to be adherent (OR = 1.7, [95%CI: 1.0-2.8]; *p* = 0.033) than patients on monotherapy. Patients who received chemotherapy showed better adherence (OR = 3.0, [95%CI: 2.2-3.9]; *p* < 0.001) than those who did not. This effect was observed for all ages (Fig. 2). Patients who received radiation therapy were also more likely to be adherent (OR = 1.8, [95%CI: 1.3-2.4]; *p* < 0.001). When adjusting for age, increasing tumour-stage was associated with a greater likelihood of adherence (OR = 1.5, [95%CI: 1.1-2.1]; *p* = 0.011) for T2-tumors and (OR = 3.7, [95%CI, 1.2-11.2]; *p* = 0.023) for T3- and T4-tumors. Furthermore, we found that after adjusting for age, those who received chemotherapy showed better adherence (OR = 3.8, [95%CI: 2.6-5.5]; *p* < 0.001).

**Table 2:** Predictors of adherence

Characteristics	OR (95% CI)	*P*-value	OR (95% CI)	*P*-value
	Unadjusted		Adjusted for age	
Age at diagnosis				
< 40	0.4 (0.2-0.7)	0.001		
40-49	1.3 (0.8-2.0)	0.306		
50-59	Ref			
60-69	0.8 (0.6-1.2)	0.230		
70-79	0.7 (0.5-1.2)	0.203		
≥ 80	0.2 (0.1-0.4)	< 0.001		
Tumour stage				
T1	Ref		Ref	
T2	1.2 (0.9-1.6)	0.258	1.5 (1.1-2.1)	0.011
T3-T4	2.5 (0.8-7.1)	0.098	3.7 (1.2-11.2)	0.023
Unknown	1.2 (0.8-2.0)	0.368	1.3 (0.8-2.1)	0.286
Histopathological type				
Ductal	Ref		Ref	
Lobular	1.3 (0.8-2.0)	0.256	1.3 (0.9-2.1)	0.188
Other	0.8 (0.5-1.4)	0.454	1.0 (0.6-1.6)	0.915
Unknown	0.8 (0.2-4.2)	0.803	1.0 (0.2-5.8)	0.964
Histological grade				
Grade 1	Ref		Ref	
Grade 2	1.0 (0.7-1.7)	0.858	1.0 (0.6-1.6)	0.992
Grade 3	1.5 (0.9-2.5)	0.124	1.6 (0.9-2.7)	0.108
Unknown	1.3 (0.7-2.4)	0.417	1.2 (0.6-2.3)	0.550
Axillary lymph node metastasis				
0	Ref		Ref	
1-3	2.1 (1.5-3.0)	< 0.001	2.2 (1.6-3.1)	< 0.001
≥ 4	2.0 (1.3-3.2)	0.004	2.1 (1.3-3.4)	0.002
Unknown	0.7 (0.4-1.2)	0.175	1.3 (0.7-2.6)	0.415
Neoadjuvant treatment				
No	Ref		Ref	
Yes	1.3 (0.9-1.9)	0.220	1.4 (0.9-2.1)	0.098
Type of antihormonal treatment				
Tamoxifen only	Ref		Ref	
Tamoxifen ➔ AI	2.4 (1.6-3.7)	< 0.001	1.6 (1.0-2.6)	0.034
AI only	2.0 (1.3-3.0)	0.001	1.5 (0.9-2.4)	0.099
AI ➔ Tamoxifen	1.9 (1.1-3.4)	0.031	1.3 (0.7-2.4)	0.484
More than one switch	2.0 (1.0-4.0)	0.060	1.3 (0.6-2.8)	0.437
No antihormonal treatment registered	0	0.996	0	0.995
Switch at two years				
No^a^	Ref		Ref	
Yes	1.7 (1.0-2.8)	0.033	1.5 (0.9-2.4)	0.141
Monotherapy	0.9 (0.6-1.3)	0.473	1.0 (0.7-1.5)	0.988
No antihormonal treatment registered	0	0.995	0	0.995
Radiation therapy				
No	Ref		Ref	
Yes	1.8 (1.3-2.4)	< 0.001	1.2 (0.9-1.7)	0.209
Chemotherapy				
No	Ref		Ref	
Yes	3.0 (2.2-3.9)	< 0.001	3.8 (2.6-5.5)	< 0.001

^a^Switch at some other point in time (+/− 6 months)

In the primary non-adherent group, 82.2% had T1 tumours compared to 56.3% in the adherent group (Table 3). Those without axillary lymph node metastasis comprised 89.1% of the PNA-group as opposed to 52.4% of the adherent group. Among patients who did not receive chemotherapy, 95.0% were PNA as opposed to 42.0% in the adherent group (p < 0.001).

**Table 3:** Characteristics of patients related to adherence

	Primary non-adherent (%)	Secondary non-adherent (%)	Adherent (%)	*P*-value (χ ^2^ Pearson’s)
Tumor stage				
T1	83 (82.2)	95 (50.5)	508 (56.3)	< 0.001
T2	14 (13.9)	67 (35.6)	275 (30.5)	
T3-T4	2 (2.0)	2 (1.1)	28 (3.1)	
Unknown	2 (2.0)	24 (12.8)	92 (10.2)	
Histological grade				
Grade 1	3 (3.0)	25 (13.3)	76 (8.4)	< 0.001
Grade 2	87 (86.1)	92 (48.9)	507 (56.1)	
Grade 3	10 (9.9)	46 (24.5)	229 (25.4)	
Unknown	1 (1.0)	25 (13.3)	91 (10.1)	
Axillary lymph node metastasis				
0	90 (89.1)	102 (54.3)	473 (52.4)	< 0.001
1-3	7 (6.9)	47 (25.0)	280 (31.0)	
≥ 4	1 (1.0)	23 (12.2)	119 (13.2)	
Unknown	3 (3.0)	16 (8.5)	31 (3.4)	
Radiation				
No	39 (38.6)	72 (38.3)	234 (25.9)	< 0.001
Yes	62 (61.4)	116 (61.7)	669 (74.1)	
Chemotherapy				
No	96 (95.0)	101 (53.7)	379 (42.0)	< 0.001
Yes	5 (5.0)	87 (46.3)	524 (58.0)	
Neoadjuvant treatment				
No	99 (98.0)	154 (81.9)	764 (84.6)	< 0.001
Yes	2 (2.0)	34 (18.1)	139 (15.4)	

**Fig. 3:**
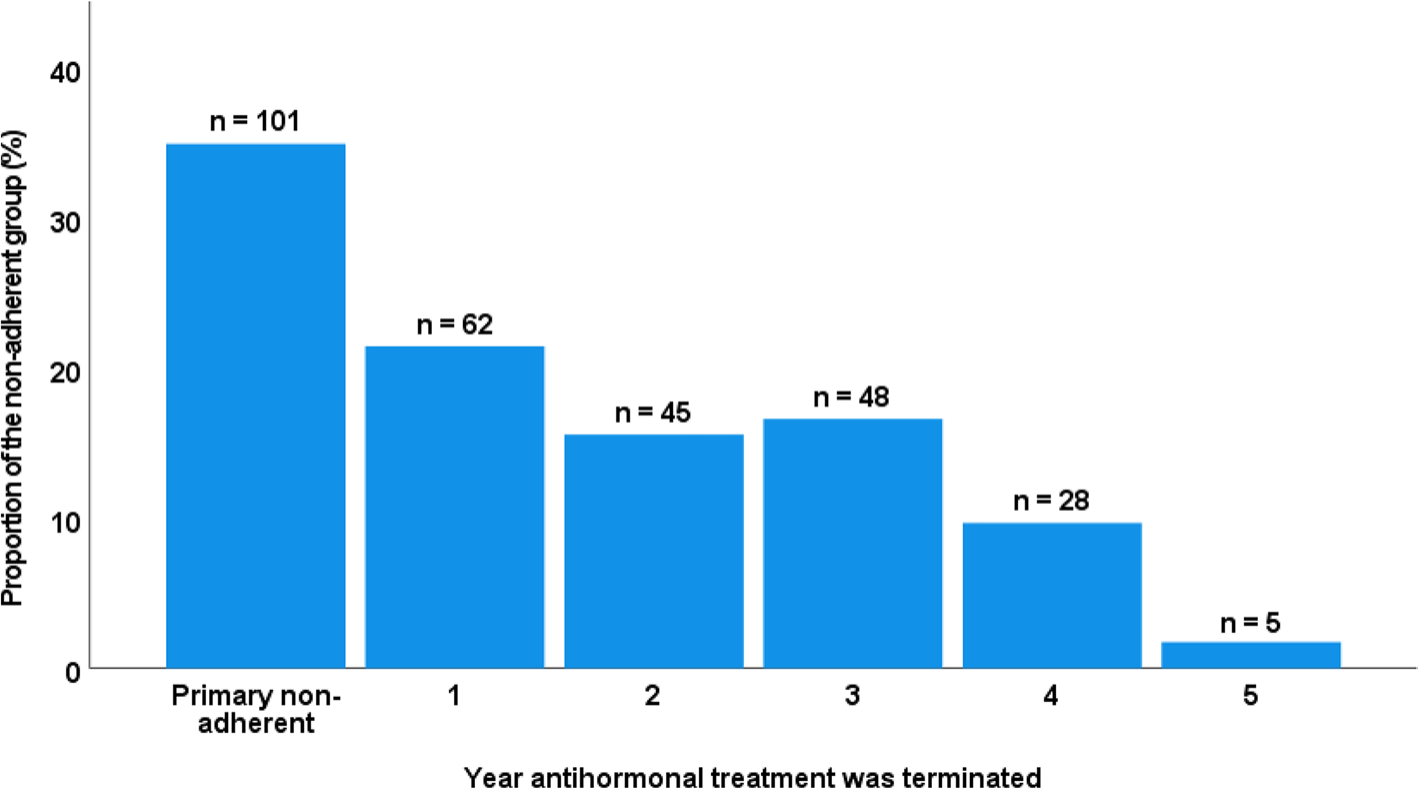
Non-adherence according to year of patient-initiated termination of treatment

For the non-adherent patients, persistence was measured as the period from initiation to termination of therapy (Fig. 3). Patients who never initiated the recommended treatment (PNA) account for 34.9% of the non-adherent patients. Furthermore, 21.5% of the patients discontinued treatment during the first year. Thereafter, 15.6, 16.6, 9.7 and 1.7% discontinued treatment during year two, three, four and five respectively.

## Discussion

In this study we found that close to a quarter of the patients (24.2%) did not adhere to prescribed treatment. Our main findings show that adherence varies with age, nodal status, type of treatment regimen and whether radiotherapy and/or chemotherapy were administered. Patients with primary non-adherence appears to have a less serious cancer-diagnosis compared to the rest of the study-population.

Poor adherence is a major challenge negatively affecting patient outcomes. It prevents medications from exerting their full beneficial effect [9–11, 14, 22]. With the use of oral medications taken at home, patients are increasingly rendered to themselves to tackle obstacles that might prevent good adherence. As antihormonal therapy in the setting of breast cancer treatment has proven to reduce the risk of recurrence and improve survival [3, 4, 8, 20, 27, 28, 40], there is no doubt that their effect is highly beneficial. This is further emphasized by the fact that current guidelines have extended the duration of treatment from five to 10 years.

A total of 659 patients (55.3%) were registered as having a MPR > 100%. These patients stayed on the antihormonal treatment beyond the recommended five-year treatment period. Some may have used antihormonal treatment for only a short period of time beyond the specified five-year recommendation. However, this finding may reflect emerging evidence at that time of the beneficial effects of antihormonal treatment given for more than five years [41–43].

The present study shows that patients aged < 40 years and ≥ 80 years are more likely to be non-adherent to antihormonal treatment. Pre-menopausal women treated with tamoxifen often develop troublesome side effects that could explain the low adherence rates in this group. Also, some of these women may have had a wish to become pregnant during the treatment period and therefore discontinued their treatment. This would be of increasing relevance with the current ten-year treatment recommendations. Patients above the age of 80 are more likely to be non-adherent due to comorbidities. These illnesses often entail complex treatment regimens. Some elderly patients suffer from varying degrees of both functional and/or cognitive impairment which further increases the risk of poor adherence [9, 44].

Our study shows that patients with metastasis to axillary lymph nodes are significantly more likely to be adherent compared to those without metastasis. This is also true when adjusting for age. Patients receiving adjuvant radiotherapy and/or chemotherapy are also significantly more likely to be adherent to antihormonal treatment than those who did not receive this treatment. These findings may reflect that more severe disease motivates patients to maintain adherence throughout the scheduled treatment period. However, most young patients receive chemotherapy and most elderly do not. In our material 96.6% of those < 40 years and 91.8% in the 40-49 year age group received chemotherapy compared to 1% ≥ 80 years and 22.5% within the 70-79 year age group. Paradoxically, despite the fact that young patients receive chemotherapy, they are more likely to non-adherent compared to older patients.

Most patients, with the exception of those with distant metastasis at the time of diagnosis, had a one-year follow-up appointment at the surgical out-patients clinic, thereafter yearly follow-up by their general practitioner for 10 years. Close follow-up has been linked to improved adherence [10, 17]. However, several studies have shown greater adherence among patients followed by an oncologist as compared to those followed by their general practitioner [23, 25, 29, 30]. As this factor is modifiable, it will be important when strategies for improving adherence are considered.

The type of treatment regimen has an impact on adherence. Some patients were recommended monotherapy and others were recommended to switch endocrine treatment (tamoxifen to AI or AI to tamoxifen) at 2 years. Our material shows significantly better adherence for those who switched at 2 years compared to those who received tamoxifen only. Some of the switches at 2 years may have been the result of side effects at the time. Although side effects are most intense in the first year of treatment, many of these patients may have continued the prescribed treatment for 2 years in the hope of fewer side effects after the planned switch at 2 years. We believe these are highly motivated patients who wish to continue the treatment despite troublesome side effects. We also observed that those who received AI as monotherapy had better adherence than those who were treated with tamoxifen monotherapy. This is in keeping with previous research [6], but is more difficult to explain but suggests that age may be a contributory factor as most women receiving AIs are postmenopausal and mainly in the age groups 60-79 years.

Of the non-adherent patients, 35.3%, or 8.5% of the total population, never initiated the treatment they were recommended. This is particularly worrisome as these patients stand to gain no benefit from the treatment. Previous research has shown that taking tamoxifen for only one to two years significantly reduces recurrence rates and breast cancer death rates [5, 45]. Motivating patients to initiate and continue their recommended treatment would therefore be advantageous in an attempt to improve adherence rates.

Of those initiating treatment, most patients with poor adherence discontinue within the first year of treatment. Thereafter, the numbers gradually decrease during the five-year treatment period. This may partly be explained by the occurrence of side effects. Research has shown that women treated with tamoxifen report fewer and less severe side effects after the first year of treatment [46]. Once the prescription is handed over to the patient, it is the patient’s responsibility to follow the recommended treatment regimen. Information about why the treatment is given, expected benefit and possible side effects are important factors to communicate to the patient in a comprehensible manner [10, 18]. Close follow-up, especially during the first year of treatment, might prevent patients from becoming non-adherent. This is exemplified by the fact that adherence often will be at its best just before and after a consultation [10, 11]. The five patients who discontinued treatment during the fifth year may have been deemed satisfactorily treated and advised to terminate their treatment as they were close to the five-year mark.

Degree of adherence may be related to certain patient characteristics. Patients with cancers with a good prognosis not warranting chemotherapy, are more frequent in the PNA-group. We failed to find a clear distinction between secondary non-adherent patients and adherent patients. The influence of other factors such as side effects and co-morbidities may have contributed to patients discontinuing their treatment earlier than recommended. However, the study of these factors was beyond the scope of this study. Our findings suggest that patients who never initiate recommended treatment have less serious cancers. A possible explanation could be that these patients fail to see the need for antihormonal treatment given their favourable diagnosis. Several factors may contribute to this misconception. Often little time is spent on explaining the importance of good adherence. In a busy clinical working day, there is often limited time available to give the patient in-depth information regarding the treatment and the prescription may be handed over to the patient with little further explanation. Important issues such as why the treatment is given, possible side effects and duration of treatment are often not adequately addressed. Clinicians may “oversell” the favourable diagnosis and prognosis to such an extent that the treatment is deemed unnecessary by the patient. Some patients may have negative expectations to antihormonal treatment [47], and this combined with a “non-serious” cancer, may increase the likelihood of not initiating the treatment. Cost of the medication is unlikely to be a contributing factor as these costs are reimbursed to the patients and do not impose a large expense. However, in some study populations, socioeconomic status may exert influence on patient adherence behaviour [48].

Our study shows that 75.8% of the patients are adherent to antihormonal treatment. This is consistent with several other studies [20, 36, 49]. We have shown that the extremes of age and less severe disease characteristics increases the risk of non-adherence. Although these are non-modifiable factors, they are important in order to identify patients at risk of poor adherence. PNA or “lack of “initiation” to antihormonal treatment has been described in previous research [23, 50, 51]. However, more in-depth knowledge about this particular group is important. In this paper we show that the PNA-patients tend to have a better prognosis and postulate that their non-initiation might be linked to how clinicians communicate. If this is the case, greater awareness among clinicians on how we communicate the need for antihormonal treatment, regardless of a good prognosis, may be a way of improving adherence rates among PNA-patients. This study has also shown that most non-adherent patients discontinue the treatment in the initial part of the treatment period, this is in keeping with previous research [38]. This finding is of value as targeted interventions for improving adherence could benefit from focusing their efforts in the initial part of the treatment period. Increased awareness of non-adherence, both among clinician and patients, will ultimately lead to better outcomes for these patients. 

The present study does not investigate subjective factors affecting adherence. Side effects are one of the major factors determining adherence to antihormonal treatment [24, 27, 29–31]. One study showed that almost half the participants reported side effects as the reason for non-adherence to tamoxifen [52]. It is important to ask patients about their experience of side effects to detect poor adherence. Prescribing ameliorative treatments for troublesome side effects will often be very beneficial [24, 29], and may prevent poor adherence. Patients’ expectations to antihormonal therapy before initiating the treatment have been proven to affect both side effects and quality of life. After 2 years of antihormonal treatment the relative risk of side effects was higher in those with negative expectations at baseline than those with low negative expectations (RR = 1.833, [95%CI: 1.0-3.3]) [47]. Patients who have been treated with chemotherapy have already been exposed to low oestrogen levels [53], this may be a contributary factor as to why these women show better adherence compared to those who did not receive chemotherapy.

Assessing adherence is challenging and complex. Several methods of measurement exist. Using prescription refill registers has been shown to be an objective and reliable way of assessing adherence. This is especially true for medications intended for long-term therapy [10, 15]. However, relying on register data, we cannot be sure that the patients consumed the medications dispensed at the pharmacy. However, we consider it unlikely that patients have repeatedly refilled their prescriptions without actually taking them. Research supports this assumption as it has been shown that the rate at which patients refill their prescriptions is consistent with the rate at which they consume their medications [54]. Furthermore, it has been shown that there is concordance between serum tamoxifen levels and rates of discontinuation [55]. Although widely used, the 80% cut-off for “good” and “poor” adherence is an arbitrary value that is poorly documented in relation to specific diseases and classes of medications [14, 56].

There are some limitations to this study. Reasons for poor adherence were not recorded. Non-adherence may be a patient choice and therefore represent intentional non-adherence. However, the decision to stop a medication may also be made by the clinician based on intercurrent illness or other factors. The unavailability of reasons for non-adherence in this study is therefore a weakness. Another drawback is the fact that data regarding antihormonal treatment prescribed to patients admitted to hospital or residing in a nursing home is not included in the data from the NorPD. The numbers in the younger and elderly subgroups are relatively small, therefore, one should be cautious in drawing firm conclusions. While some studies have shown similar adherence-rates in relation to age [29, 35], others have shown this association only for the younger age group [20, 24, 25]. A potential limitation of this study is whether the results are transferable to other patient populations. Study population profiles vary with regard to demographics, access to treatment and public health systems. However, we believe that our results contribute to our understanding of patient adherence.

Strengths of this study include its population-based design including all women that underwent surgery at St Olav’s Hospital during the study period. We have collected prescription data from the NorPD rather than relying on self-reported data from the patients. Data from prescription databases have been shown to be superior to self-reported adherence data [54] and are particularly advantageous for the evaluation of medications intended for long-term therapy [15]. Compared to many other studies with shorter periods of follow-up [28, 34, 38, 57–60], we have evaluated adherence for the full length of the recommended five-year treatment period. Furthermore, we have differentiated between treatment with tamoxifen and AIs.

## Conclusion

We conclude that adherence to antihormonal therapy for breast cancer is suboptimal. Special attention should be paid to the young and elderly as these subgroups show poorer adherence than the other age groups. The relatively large group of patients with primary non-adherence is of particular interest. It appears that these patients have cancers with a relatively good prognosis. This is a somewhat surprising finding, and we hope future research will focus more on this group of patients. Non-adherent patients tend to terminate their antihormonal therapy in the initial part of the treatment period. This means that targeted interventions to improve adherence should be focused on the initial part of the treatment period. Better adherence will improve patient outcomes and reduce health care expenditures.

## Data Availability

The datasets generated during and/or analysed during the current study are not publicly available due to reasons of sensitivity and limitations imposed in the conditions for approval by the Ethics Committee but are available from the corresponding author on reasonable request.

## Abbreviations

HR: Hormone receptor
MPR: Medical Possession ratio
AI: Aromatase inhibitor
NorPD: Norwegian Prescription database
OR: Odds ratio
CI: Confidence interval
